# O serotype-independent susceptibility of *Pseudomonas aeruginosa* to lectin-like pyocins

**DOI:** 10.1002/mbo3.210

**Published:** 2014-09-16

**Authors:** Maarten G K Ghequire, Jozef Dingemans, Jean-Paul Pirnay, Daniel De Vos, Pierre Cornelis, René De Mot

**Affiliations:** 1 Centre of Microbial and Plant Genetics, University of Leuven3001, Heverlee, Belgium; 2 Research Group Microbiology, Department of Bioengineering Sciences, Vrije Universiteit Brussel1050, Brussels, Belgium; 3 Department of Structural Biology, VIB1050, Brussels, Belgium; 4 Laboratory for Molecular and Cellular Technology, Queen Astrid Military Hospital1120, Brussels, Belgium

**Keywords:** Bacteriocin, lipopolysaccharide, LlpA, MMBL lectin, O-specific antigen

## Abstract

Lectin-like bacteriocins of the LlpA family, originally identified in plant-associated bacteria, are narrow-spectrum antibacterial proteins composed of two tandemly organized monocot mannose-binding lectin (MMBL) domains. The LlpA-like bacteriocin of *Pseudomonas aeruginosa* C1433, pyocin L1, lacks any similarity to known *P. aeruginosa* bacteriocins. The initial interaction of pyocin L1 with target cells is mediated by binding to d-rhamnose, present in the common polysaccharide antigen of lipopolysaccharides (LPS), but the actual cytotoxic mechanism is unknown. In this study, we characterized the activity range of pyocin L1 and two additional L pyocins revealed by genome mining, representing two highly diverged LlpA groups in *P. aeruginosa*. The recombinant proteins exhibit species-specific antagonistic activities down to nanomolar concentrations against clinical and environmental *P. aeruginosa* strains, including several multidrug-resistant isolates. The overlap in target strain spectrum between two close homologues of the pyocin L1 group is only minimal, contrasting with the considerable spectral redundancy of LlpA proteins reported for other *Pseudomonas* species. No correlation was found between L pyocin susceptibility and phylogenetic relatedness of *P. aeruginosa* isolates. Sensitive strains were retrieved in 13 out of 15 O serotypes tested, excluding the possibility that the highly variable and immunogenic O serotype antigen of the LPS coating would represent a dominant susceptibility-discriminating factor.

## Introduction

*Pseudomonas aeruginosa* is a ubiquitous environmental *γ*-proteobacterium and an opportunistic human pathogen. Its metabolic versatility allows dwelling in diverse niches, including plant rhizospheres and aqueous environments. In clinical settings, biofilm formation by *P. aeruginosa* is associated with enhanced levels of antibiotic resistance and chronic, difficult-to-treat infections (Ryder et al. 2007) and the organism is of high risk to immunosuppressed, burn wound, and cystic fibrosis patients (Church et al. 2006; Lipuma 2010). The increasing prevalence of multidrug-resistant *P. aeruginosa* urgently requires novel antimicrobials with alternative molecular targets to be developed. One possible strategy would be to exploit the arsenal of natural compounds involved in intra-species competition (Allen et al. 2014).

*Pseudomonas aeruginosa* bacteriocins, designated pyocins, are potent species-specific killing agents. R and F pyocins are phage tail-like devices (also designated tailocins), whereas S and M pyocins are modular proteins degrading nucleic acids or affecting cell envelope integrity (Ghequire and De Mot 2014). A novel pyocin type unrelated to the former classes was recently identified in a strain isolated from a cystic fibrosis patient (McCaughey et al. 2014). Pyocin L1 (PyoL1) belongs to the LlpA family of lectin-like bacteriocins that are composed of a monocot mannose-binding lectin (MMBL) domain tandem. LlpA bacteriocins were identified in nonpathogenic rhizosphere isolates of *Pseudomonas putida* (Parret et al. 2003) and *Pseudomonas protegens* (Parret et al. 2005), phytopathogens *Pseudomonas syringae* and *Xanthomonas citri* (Ghequire et al. 2012b), and the human pathogen *Burkholderia cenocepacia* (Ghequire et al. 2013b).

LlpA proteins display structural similarities to plant MMBL lectins, but their mannose-binding capacity is low (Ghequire et al. 2013a; McCaughey et al. 2014). Instead, affinity for d-rhamnose is much higher and its presence as a homopolymeric constituent of the common polysaccharide antigen (CPA or A-band) of *P. aeruginosa* (Lam et al. 2011) mediates binding of PyoL1 to lipopolysaccharide (LPS) of target cells (McCaughey et al. 2014). Whereas carbohydrate binding by LlpA has only been attributed to the carboxy-terminal MMBL domain (Ghequire et al. 2013a; McCaughey et al. 2014), domain exchanges between *P. putida* and *P. protegens* LlpAs demonstrated that the amino-terminal MMBL domain is a major determinant of target strain specificity (Ghequire et al. 2013a). However, the molecular mechanism engendering cytotoxicity remains elusive.

The killing range and potency of PyoL1 among pseudomonads has not yet been studied. We compared the activity and target spectra of two additional L-type pyocins, representative for the LlpA diversity in *P. aeruginosa* genomes, to those of PyoL1. These results exclude O-specific antigen (OSA) repeat units of LPS as a candidate sensitivity-discriminating factor of L pyocins and suggest a different, highly variable molecular determinant to be involved in target strain specificity.

## Materials and Methods

### Strains and culture conditions

*Escherichia coli* was grown in Lysogeny Broth (LB, MP Biomedicals, Brussels, Belgium) at 37°C. *Pseudomonas* strains (Table S1) were routinely grown in Trypticase Soy Broth (TSB, BD Biosciences, Erembodegem, Belgium) at 30°C, except for *P. aeruginosa* that was cultured in LB at 37°C. Media were solidified with 1.5% agar (Invitrogen, Merelbeke, Belgium) and supplemented with 50 *μ*g/mL kanamycin (Sigma-Aldrich, Diegem, Belgium) when required.

*Escherichia coli* TOP10F' (Invitrogen) was used for the propagation of plasmids for sequencing (GATC Biotech, Constance, Germany) and *E. coli* BL21(DE3) (Novagen, Darmstadt, Germany) for expression of pyocin genes (Integrated DNA Technologies, Haasrode, Belgium). Plasmid DNA was extracted using the QIAprep Spin Miniprep Kit (Qiagen, Venlo, Netherlands).

### Recombinant DNA methods

Standard methods were used for preparing competent *E. coli* cells and heat shock transformation. Restriction enzymes (Roche Diagnostics, Vilvoorde, Belgium) and T4 DNA ligase (Invitrogen) were used according to the supplier's specifications.

Pyocin genes were amplified from synthetic DNA (IDT DNA) by PCR with PfuUltra II Fusion HS DNA polymerase (Agilent Technologies, Diegem, Belgium), using a C1000 Thermal Cycler (Bio-Rad, Temse, Belgium). Primers are listed in Table S2. Amplicons were purified using the QIAquick PCR purification Kit (Qiagen), digested with NdeI and XhoI, ligated in pET28a(+) (Novagen), and transformed to *E. coli* TOP10F' cells. Transformants were insert verified by PCR using Taq polymerase (BIOKÉ, Leiden, Netherlands) with primers PGPRB-10029 and PGPRB-10030. Confirmed plasmids pCMPG6209 (encoding PyoL1 from *P. aeruginosa* C1426), pCMPG6210 (pyocin L2 (PyoL2) from *P. aeruginosa* 62) and pCMPG6211 (pyocin L3 (PyoL3) from *P. aeruginosa* BWHPSA007) were verified by sequencing. Pyocin genes were cloned with an amino-terminal His_6_-tag, and in the case of PyoL3 without its predicted amino-terminal secretory signal peptide sequence (SignalP 4.1, http://www.cbs.dtu.dk/services/SignalP/).

### Overexpression and purification of recombinant pyocins

Induction of expression, lysis of harvested cells by sonication, protein isolation, and purification of His_6_-tagged PyoL1, PyoL2 and PyoL3 from *E. coli* BL21(DE3) were performed as described previously (Parret et al. 2004). Contaminating proteins from the eluted fractions were removed via gel filtration on a Superdex 200 column 16/60 (Amersham Biosciences, Diegem, Belgium), run at 1 mL/min with bis-tris propane buffer (20 mmol/L, NaCl 200 mmol/L, pH 7.0). Concentrations of purified recombinant proteins were determined by absorbance measurement at 280 nm with molar extinction coefficients of 63,370 mol/L/cm for PyoL1 (calculated molecular weight 30,445 Da), 61,880 mol/L/cm for PyoL2 (30,445 Da) and 64,985 mol/L/cm for PyoL3 (31,945 Da).

### Bacteriocin spot assay

Antagonistic activity of purified recombinant pyocins was verified by spot assay as described previously (Ghequire et al. 2012b). Briefly, 10-*μ*L spots of purified pyocin (1 mg/mL or a serial dilution thereof) were applied on a cell lawn of the *Pseudomonas* strain of interest. Bis-tris propane buffer was used as a negative control. After spot drying, plates were incubated overnight at 30°C or 37°C and evaluated for the presence of halos next day.

### Determination of pyocin MICs

Minimum inhibitory concentrations (MICs) of the pyocins were determined using a Bioscreen C apparatus (Oy Growth Curves Ab Ltd, Helsinki, Finland) as described previously (Ghequire et al. 2013a). The MIC was defined as the minimum concentration of protein at which no growth of the indicator strain was observed (OD_600_ < 0.1) after 24 h. At least three independent repeats, with three replicates each, were carried out.

### Pyocin L2 modeling

A PyoL2 model was generated using I-TASSER (Roy et al. 2010) (http://zhanglab.ccmb.med.umich.edu/I-TASSER). Structure analysis and visualization were performed with PyMOL.

### Phylogenetic analysis

*Pseudomonas aeruginosa* tandem-MMBL proteins were retrieved by homology searches (BlastP) with known *Pseudomonas* LlpA sequences as queries. Sequence alignments and phylogenetic analyses were performed with Geneious 7.1.2 (http://www.geneious.com). Predicted amino-terminal signal peptide sequences, if present, were removed before alignment.

### Combined data analysis

A previously compiled data set (Pirnay et al. 2009) was analyzed for the strains used as indicators with BioNumerics v6.8. Feature comparison of individual strains resulted in individual similarity matrices that were averaged into the similarity matrix of the composite data set. Each isolate corresponds to a polyphasic profile that contributed. The composite similarity matrix was grouped by minimum spanning tree (MST) analysis, as described previously.

## Results and Discussion

### Two distinct groups of tandem-MMBL proteins occur in *P. aeruginosa*

**Figure 1:**
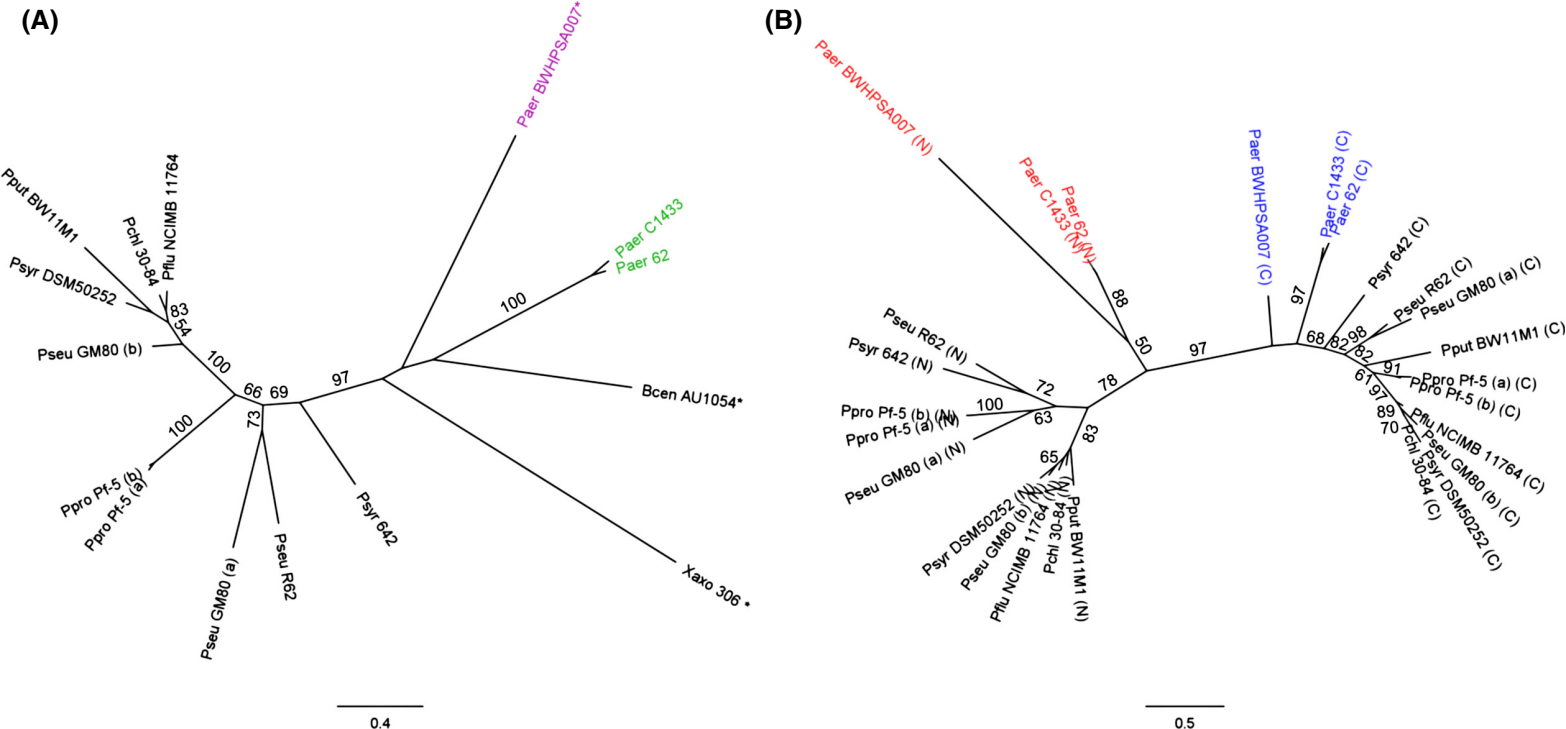
Phylogeny of *Pseudomonas aeruginosa* proteins with tandem-monocot mannose-binding lectin (MMBL) architecture. (A) Unrooted maximum-likelihood tree inferred from a multiple amino acid sequence alignment (Fig. S1) of bacteriocins from *P. aeruginosa* C1433, *Pseudomonas putida* BW11M1, *Pseudomonas protegens* Pf-5 (a, PFL_1229; b, PFL_2127), *Pseudomonas syringae* pv. *syringae* 642, *X. axonopodis* pv. *citri* str. 306, *B. cenocepacia* AU1054, with hypothetical proteins from *P. aeruginosa* BWHPSA007 (ERZ04935), *P. aeruginosa* 62 (ERX71449), *P. chlororaphis* 30-84 (EJL08681), *Pseudomonas fluorescens* NCIMB 11764 (ERP40244), *P. syringae* DSM50252 (EGH77666), *Pseudomonas* sp. GM80 (a, EJN36166; b, EJN36323) and R62 (derived from AHZM01000533). Orthologues sharing >95% amino acid identity with one of the listed proteins are not included in the tree and Figure S1. Predicted amino-terminal signal peptide sequences were removed before alignment (names marked with asterisk). Pairs of tandem MMBL proteins present in a particular strain are indicated with extensions (a) and (b). The scale bar represents 0.4 substitutions per site. The positions of the C1433 and BWHPSA007 pyocin groups are highlighted in green and purple, respectively. (B) Unrooted maximum-likelihood tree inferred from a multiple amino acid sequence alignment of individual MMBL modules extracted from *Pseudomonas* tandem-MMBL proteins (Fig. S1). Protein sequences and designations are the same as used in (A). The *P. aeruginosa* amino-terminal (N, red) and carboxy-terminal-domains (C, blue) are highlighted. The scale bar represents 0.5 substitutions per site. Bootstrap values (percentage of 100 replicates) higher than or equal to 50 are shown at the branches.

Multiple genes encoding proteins with a tandem-MMBL domain architecture were retrieved in several *P. aeruginosa* genomic sequences. Being only distantly related to LlpAs of other *Pseudomonas* species (<30% amino acid sequence identity), these proteins fall into two remote groups with borderline pairwise homology (˜28% identity) (Fig. 1A, Fig. S1, Table S3). One cluster consists of PyoL1 homologues with sequences either identical to the C1433 protein (in strain C1426, the non-mucoid progenitor of mucoid strain C1433) (Stewart et al. 2014) or sharing >86% identity to it (in strains 62, 3578, 3579, BWHPSA001, and BWHPSA016; *Pseudomonas aeruginosa* initiative, Broad Institute). Within the newly identified second cluster, pairwise sequence identities between the proteins from strains BK1 (Jeganathan et al. 2014), BWHPSA007 (with two homologues) and BWH033 (*Pseudomonas aeruginosa* initiative, Broad Institute), and VRFPA06 (Murugan et al. 2014) exceed 95%. The members of the respective clusters also differ by the presence (BWHPSA007 group) or absence (C1433 group) of an amino-terminal Sec-specific signal sequence. In this respect, PyoL1 and its homologues resemble other *Pseudomonas* LlpAs, lacking a discernable secretion motif, whereas the members of the second group are most likely substrates of the Sec translocon, similarly to *Xanthomonas* and *Burkholderia* LlpAs (Ghequire et al. 2012b, 2013b).

The genomic regions carrying the pyocin L-like genes exhibit extended sequence identity between strains C1433 and its progenitor C1426, but quite different genomic contexts are apparent in the other strains. The BWHPSA007 genomic region encoding Q020_03570 is largely conserved in strains BK1 and VRFPA06 but lacks synteny in other strains. Remarkably, the BWH033 gene encoding AJ73_04554, an identical copy of Q020_03570, is found in a different genomic context, that in turn is highly similar to the phage-like region encoding Q020_01808, the second L-type pyocin of strain BWHPSA007 that differs by 9 out of 290 residues from the Q020_03570 amino acid sequence. The presence of adjacent prophage genes emerges as a common feature and is reminiscent of the presence of *llpA* cargo genes on prophages in *Pseudomonas* plant isolates (Ghequire and De Mot 2014). In strain 62, the pyocin-carrying prophage is located between the *mutS* and *cinA* genes, the same intergenic region as targeted by prophages loaded with *llpA* genes in *P. protegens* Pf-5 and *Pseudomonas* sp. GM80. This stretch is equally a hotspot for insertion of tailocins in non-*P. aeruginosa* strains, whereas the different *P. aeruginosa* R and F pyocins are consistently located between the *trpE* and *trpG* genes (Ghequire and De Mot 2014).

The phage-associated contexts of lectin-like bacteriocin genes suggest acquisition by horizontal transfer of plant-derived MMBL lectin genes that subsequently evolved toward new functionalities as antibacterials. Hitherto, *llpA* genes were only found in plant-associated and soil-dwelling pseudomonads. The *P. aeruginosa* strains carrying *llpA*-like genes all originate from human sources (abscess, blood, CF lung, cornea, ear, urine). In general, clinical isolates are also well adapted to live in soil and rhizospheres.

**Figure 2:**
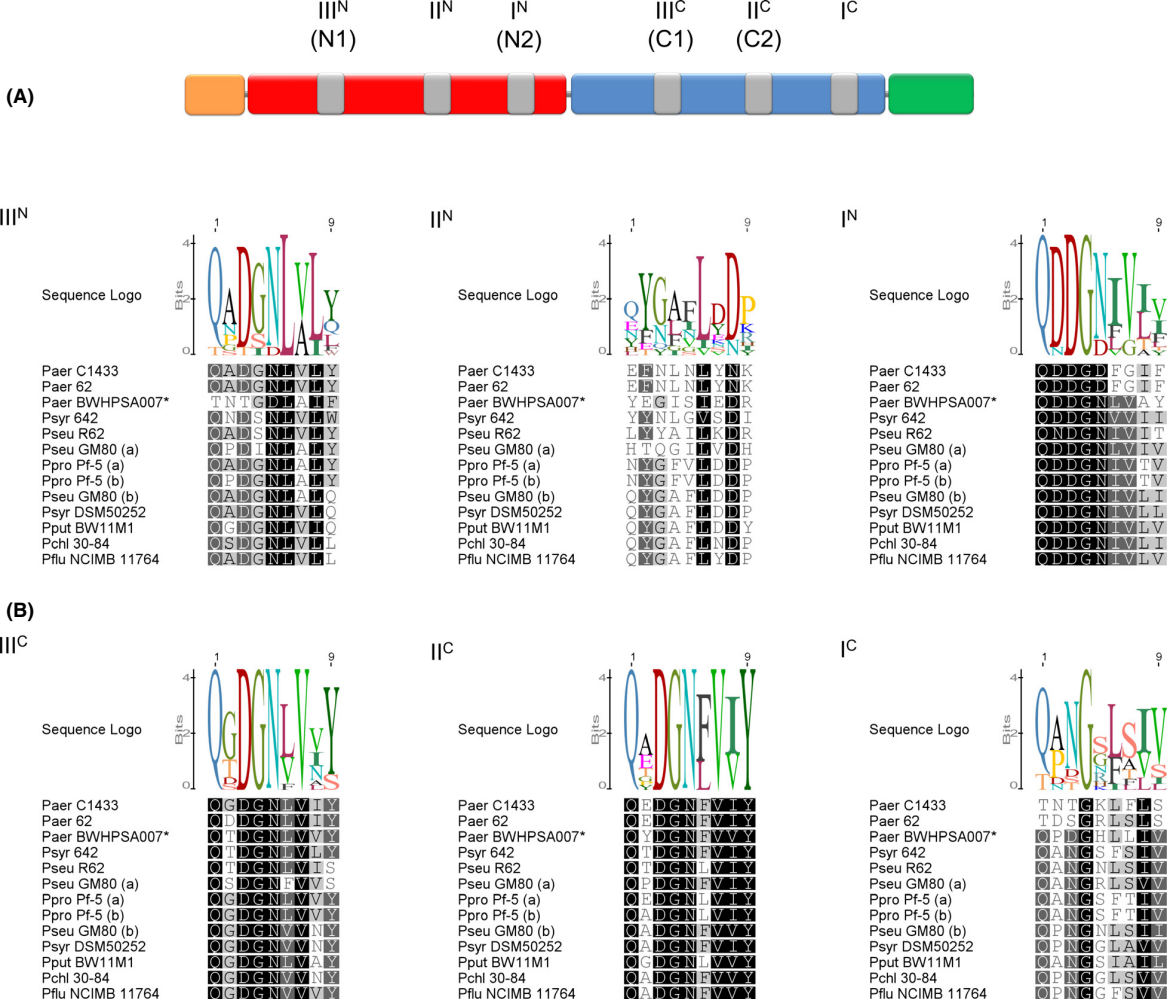
Domain structure of L pyocins (A) and carbohydrate-binding sites in pseudomonad tandem-monocot mannose-binding lectin (MMBLs) (B). (A) The cleavable secretion signal sequence, if present, is indicated by an orange box. The amino-terminal MMBL domain is colored red, the carboxy-terminal MMBL domain blue and the C-terminal extension green. The six candidate carbohydrate-binding sites, with their corresponding designations, are marked by gray boxes. Alternative site names are indicated between parentheses (McCaughey et al. 2014). (B) Sequence alignments of the carbohydrate-binding sites corresponding to the consensus motif QxDxNxVxY in LlpAs and L pyocins (derived from sequence alignment in Fig. S1) are shown in differential shading, corresponding to sequence conservation. The sequence logo graph visualizes the degree of consensus for each residue. Sequence designations are specified in the legend of Figure 1.

The MMBL domains within each *P. aeruginosa* tandem display pronounced sequence divergence (<28% amino acid identity; Fig. 1B), similar to previously characterized LlpAs in *Pseudomonas* (Ghequire et al. 2012a). This consistent domain segregation reflects a functional specialization of the MMBL modules and experimental evidence in support of this was reported for *P. putida* and *P. protegens* LlpAs (Ghequire et al. 2013a). The QxDxNxVxY motifs defining the rhamnose-binding pockets III^C^ and II^C^ of pyoL1 are well conserved across both *P. aeruginosa* groups, suggesting a similar binding potential of the carboxy-terminal MMBL domain (Fig. 2). However, at sites I^C^, I^N^, and III^N^, the C1433 and BWHPSA007 groups carry quite dissimilar sequences. While the III^N^ motif of the C1433 group resembles well the corresponding sequences of non-*P. aeruginosa* LlpAs, the reverse is true for the I^N^ and I^C^ motifs that are better preserved in BWHPSA007. The functional implications of this apparent group-linked motif differentiation are not clear. Sites I^N^ and I^C^ are not surface-exposed, excluding their involvement in the carbohydrate-binding function of the bacteriocin, and the weak binding to site III^N^ is probably of little physiological significance, if any (Ghequire et al. 2013a; McCaughey et al. 2014). The highly degenerate II^N^ site likely lost its carbohydrate-binding potential completely in all pseudomonads.

### Potent species-specific antagonistic activities of L pyocins

**Figure 3:**
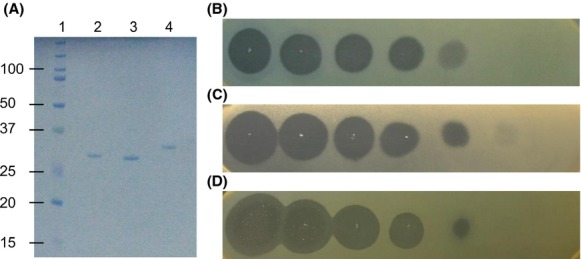
Purification and activity of L pyocins. (A) SDS-PAGE electrophoresis of purified pyocins. Lane 1, size marker (kDa); lane 2, PyoL1; lane 3, PyoL2; lane 4, PyoL3. (B–D) Bacteriocin activity of a 5-fold dilution series (starting concentration 2 mg/mL, left halo) of PyoL1 (B), PyoL2 (C) and PyoL3 (D) against indicator strains *Pseudomonas aeruginosa* Bu002 (B), A19 (C) and Pr335 (D).

In addition to PyoL1, its most diverged group member, protein P997_04049 (PyoL2 from strain 62) and protein Q020_01808 (PyoL3 from strain BWHSPA007) as a representative for the second cluster (Fig. 1A), were cloned in pET28(a) and expressed in *E. coli* BL21(DE3). For convenient purification, a sequence encoding a His_6_-tag was incorporated at the 5′-end of the respective pyocin coding regions since amino-terminal hexa-His fusions have no detectable effect on activity or specificity of recombinant LlpA proteins, contrary to carboxy-terminal fusions in some cases (Parret et al. 2004; Ghequire et al. 2012b). For Q020_01808, the tag was immediately fused to the predicted mature protein without signal peptide. Recombinant proteins were purified by affinity chromatography on Ni-NTA and polished by gel filtration. The calculated molecular weights of PyoL1 (30,445 Da), PyoL2 (30,445 Da), and PyoL3 (31,945 Da) match well with the apparent sizes of the respective recombinant proteins as estimated by SDS-PAGE: 29.0, 28.1 and 30.9 kDa, respectively (Fig. 3A).

**Figure 4:**
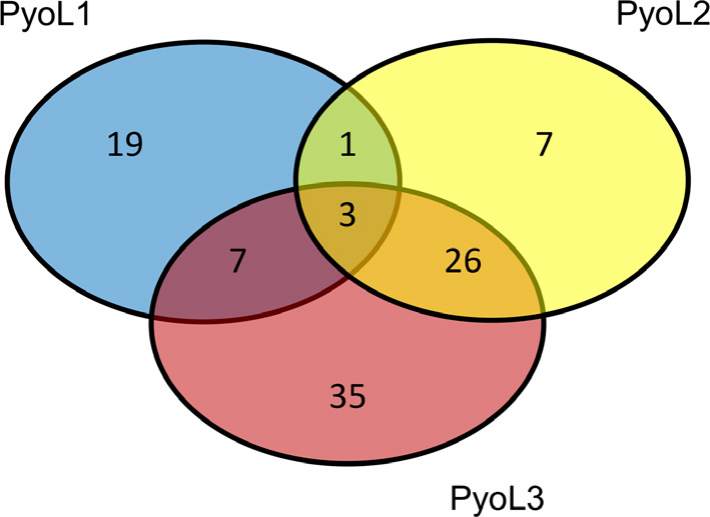
Venn diagram describing L pyocin susceptibilities of 150 *Pseudomonas aeruginosa* strains. About one-third of strains (52) in the test panel was not inhibited by PyoL1, PyoL2 or PyoL3.

Antibacterial activity of the purified proteins was assessed against a panel of clinical and environmental *P. aeruginosa* isolates and several strains of other *Pseudomonas* species. For this purpose, drops of recombinant protein were spotted onto *Pseudomonas* cell lawns, grown overnight at 30°C or 37°C, and scored the following day for growth inhibition halos (Fig. 3B, C, and D). A major number of the *P. aeruginosa* isolates proved pyocin L susceptible, while no activity was observed against other *Pseudomonas* species (Table 1, Table S1). More than 65% of the tested *P. aeruginosa* strains were susceptible to at least one pyocin, with 20% being killed by PyoL1, 24.7% by PyoL2, and 47.3% by PyoL3 (Fig. 4). These values are higher than those of LlpAs in other species, typically targeting only 10–15% of the respective test panels (Ghequire et al. 2012b, 2013b). Interestingly, several susceptible strains targeted by these pyocins are known to be multidrug-resistant (Table S1). MICs of the most sensitive strains are within the lower nanomolar range (Table 2). These values are of the same order of magnitude as the MIC of *P. putida* BW11M1 LlpA against *P. syringae* GR12-2R3 (2.08 nmol/L) (Ghequire et al. 2013a). The MICs of L pyocins are comparable to inhibitory concentrations reported for modular pyocins, such as pyocin S5 from strain PAO1 (Ling et al. 2010) and the M-type pyocins PaeM (Barreteau et al. 2009) and syringacin M (Grinter et al. 2012).

**Table 1:** Susceptibility of *Pseudomonas aeruginosa* strains with different O serotypes and of strains from other *Pseudomonas* species to three L pyocins

Species	O serotype	# strains tested	PyoL1	PyoL2	PyoL3
*Pseudomonas aeruginosa*	O1	7	1	2	5
	O2	2	0	0	2
	O3	4	0	2	3
	O4	2	1	1	1
	O5	2	1	0	0
	O6	9	2	2	6
	O7	1	1	0	0
	O8	3	0	2	0
	O9	3	0	1	2
	O10	2	0	0	1
	O11	17	9	0	3
	O12	9	0	0	1
	O15	1	0	0	0
	O17	1	0	1	0
	O18	1	0	0	0
	NT	86	15	26	47
*Pseudomonas fluorescens*		34	0	0	0
*Pseudomonas putida*		9	0	0	0
*Pseudomonas savastanoi*		15	0	0	0
*Pseudomonas syringae*		10	0	0	0
*Pseudomonas tolaasii*		8	0	0	0
*Pseudomonas* spp.		16	0	0	0
***Total***		***242***	***30***	***37***	***71***

A list of strains tested and their individual sensitivity profiles is shown in Table S1. NT, polyagglutinable or not serotyped.

**Table 2:** Minimum inhibitory concentrations of PyoL1, PyoL2, and PyoL3 for selected *Pseudomonas aeruginosa* strains

	MIC (nmol/L)		
Strain	PyoL1	PyoL2	PyoL3
Br776	26.0	–	–
Bu007	10.8	>3300	–
CFPA13	–	13.8	6.9
CFPA22	>3300	61.8	77.8
CFPA54	–	–	19.4
CFPA87	–	–	55.0
CFPA101	–	>3300	>3300
CFPA118	–	37.0	–
CFPA120	–	61.8	9.7
CFPA124	–	61.8	13.8
CPHL 6750	–	46.2	–
LMG 1272	88.1	–	–
PA134	13.4	–	–
PA135	30.0	–	–
PA229	224.5	–	–
PAO1	38.3	–	–

Three strains were killed by all three L-type pyocins. Interestingly, the spectrum overlap between PyoL1 and PyoL2 is only minimal, despite their high sequence identity (Table S3). Only four out of 63 sensitive strains proved susceptible to both PyoL1 and PyoL2, compared to a proportion of 10/91 for PyoL1 and PyoL3 and 29/79 for PyoL2 and PyoL3 (Fig. 4)_._ Moreover, a pronounced quantitative difference in sensitivity to the related bacteriocins PyoL1 and PyoL2 was noted in case of such dual susceptibility (>50-fold difference in MIC values; Table 2). This observation was unexpected, given the extensively overlapping susceptibility profiles reported for LlpA proteins from pseudomonad species *P. putida*, *P. protegens*, and *P. syringae,* that share <50% amino acid sequence identity (Ghequire et al. 2012a). Furthermore, no strain with different susceptibility to the orthologues LlpA1 and LlpA2 (94% identity) encoded by *P. protegens* Pf-5 could be identified (Parret et al. 2005).

**Figure 5:**
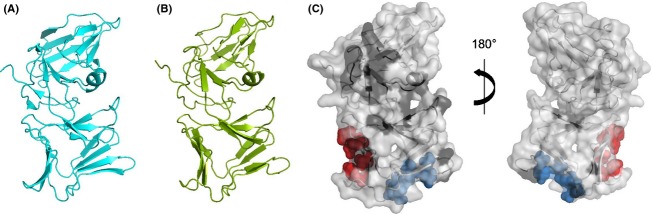
Structural diversity of L pyocins. (A) Cartoon representation of PyoL1 from *Pseudomonas aeruginosa* C1433 (PDB 4LE7). (B) Structural model of PyoL2 from strain 62. Estimated accuracy of the model is 1.42 (C-score) with a template modeling score of 0.91 ± 0.06 and root mean square deviation of 3.0 ± 2.2 Å. (C) Cartoon-surface representations of the PyoL2 model. Residues constituting the D-rhamnose binding sites III^C^ and II^C^ are shown as blue and red spheres, respectively. Nonconserved residues differing between PyoL2 and PyoL1 are indicated in black. Conservative substitutions are not marked: V-M, E-Q, G-A, I-L, I-V, A-V, R-K, N-D, T-S.

The pronounced sequence homology between PyoL1 and PyoL2 (86% amino acid identity) enabled to generate a reliable model for PyoL2 (Fig. 5B) based on the PyoL1 structure (Fig. 5A) (McCaughey et al. 2014). Nonconservative amino acid substitutions between the PyoL1 and PyoL2 sequences predominantly cluster on one side of the amino-terminal MMBL domain (Fig. 5C). These patches on PyoL1 and PyoL2 are good candidate interaction sites mediating differential activity toward susceptible strains.

Based on currently available data, a model is proposed in which CPA on target cells serves as a primary receptor recognized by the carboxy-terminal MMBL domain of L-type pyocins. To engender actual killing of CPA-selected potential targets, the amino-terminal domain would need to recognize an as yet unknown strain-specific secondary interaction partner, resulting in the observed target specificity. This model is reminiscent of the two-stage interaction of separate bacteriocin domains with pairs of outer membrane components that mediates entry of most colicins into targeted *E. coli* cells (Jakes 2014). After initial docking onto a plugged *β*-barrel protein or, alternatively, after binding to LPS as a primary receptor in case of colicin N (Johnson et al. 2014), subsequent interaction with a nearby porin triggers uptake of the toxin.

### Susceptibility of L pyocins is O-serotype independent

**Figure 6:**
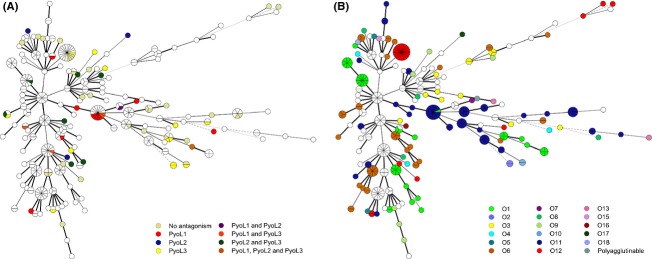
Functional diversity of L pyocins. Tree representation inferred from the similarity matrix of a composite data set of 328 *Pseudomonas aeruginosa* strains, marking L pyocin susceptibility (A) and distribution of O serotypes (B). The respective color legends specify pyocin susceptibility (A) and O serotype (B). White dots represent untested (A) or untypable strains (B). Circles correspond to a polyphasic profile and are scaled with an increasing number of member representatives. Line color and style represent branch lengths. A decreasing phylogenetic relatedness corresponds to a gradual decline in line style, from solid to dashed and color change, from black to gray, respectively. The grouping of the resulting averaged composite similarity matrix was achieved by standard MST analysis, with single and double locus variable priority rules. Priority standards were as follows: identification policy, all taxa with zero inter-taxon distance were identified; priority rule 1, maximum number of N-locus variants (*N* = 1) with weight 10000; priority rule 2, maximum number of N-locus variants (*N* = 2) with weight 10. Branch length scaling is logarithmic. Line color and style represent branch lengths: lengths up to 1, thickness 2 and solid; lengths up to 3, thickness 1 and solid; lengths up to 4, thickness 1 and dashed; lengths above 4, thickness 1 and dotted. Line color changes gradually from black (up to 1) to light gray (above 4).

Seventy strains in the test panel were selected from a collection of 328 *P. aeruginosa* isolates, based on their distribution across a MST previously built from a polyphasic data set (Pirnay et al. 2009). Characteristics include O serotype, fluorescent amplified-fragment length polymorphism pattern, gene sequences of outer membrane proteins (*oprI, oprL*, and *oprD*), pyoverdine receptors (*fpvA* and *fpvB*), group I pilin glycosyltransferase (*tfpO*), and the prevalence of exoenzyme genes (*exoS* and *exoU*). Patterns of pyocin sensitivity did not correlate with any of these characteristics, as variation occurred even within clonal complexes (Fig. 6A). Susceptible strains were found in virtually all MST clusters and, hence, liability to pyocin L-mediated killing cannot be attributed to phylogenetic relatedness, suggesting that (a) highly variable epitope(s) mainly determine(s) strain victimization.

One of the most variable and immunodominant surface-exposed epitopes on the *P. aeruginosa* cell envelope is the OSA, formerly termed the B-band (Lam et al. 2011). The indicator strains with a known O serotype occupy essentially random positions along the tree, underscoring the highly strain-specific nature of this cell surface property (Fig. 6B). No correlation seems to exist between strain susceptibility and O serotype (Table 1). The test panel covers 15 O serotypes (64 strains with known O serotype, excluding polyagglutinable strains), of which 13 proved susceptible to at least one of the three lectin-like pyocins. The actual number of serotypes harboring sensitive strains may even be higher since the test panel contained only one strain for both O serotypes denoted as insensitive. These findings rule out the OSA as a susceptibility-discriminating factor and suggest that such a role may be played by another variable surface characteristic, possibly linked to the as yet undisclosed secondary interaction partner.

In *P. aeruginosa* PAO1, CPA appears to play a role in biofilm formation since a CPA-lacking mutant is not able to develop robust biofilms (Murphy et al. 2014). Hence, exploiting CPA as a bacteriocin receptor may represent a cunning strategy to initiate attack with a L-type pyocin on competing with *P. aeruginosa* strains in a mixed biofilm. According to this hypothesis, subject to experimental verification, a pyocin L-producing strain would be able to kill CPA-proficient susceptible rivals in such a biofilm, while CPA-deficient mutants trying to escape from the bacteriocin attack would be impaired in their biofilm-forming capacity. It will be of interest to further elucidate the molecular basis of the lethal activity engendered by lectin-like pyocins, as identification of the ultimate cellular target may pave the way for the design of novel, highly selective antibacterials.

## Supporting Information

Figure S1 Multiple amino acid sequence alignment used to construct the phylogenetic tree of prokaryotic tandem-monocot mannose-binding lectin (MMBL) homologues (Fig. 1A). Sequence conservation is visualized by differential shading. The locations of MMBL modules in the amino-terminal domain (MMBL-N) and the carboxy-terminal domain (MMBL-C) are indicated by yellow boxes along the consensus sequence. The positions of the MMBL motifs constituting (putative) sugar-binding sites are marked by purple boxes. Aligned MMBL modules were used for the construction of the ML tree of Figure 1B (using the JTT matrix). Sequence designations are provided in the legend of Figure 1.

Table S1 List of *Pseudomonas* strains used in this study and patterns of susceptibility to the different L-type pyocins tested. ABR: antibiotic resistance is expressed as the number of antibiotic classes to which resistance was detected (Pirnay et al. 2009. PLoS One 4, e7740). NT, not tested.

Table S2 List of primers used in this study.

Table S3 Percentages of pairwise sequence identities between the sequences of prokaryotic tandem-monocot mannose-binding lectin (MMBL) proteins shown in Figure 1A. Strain information is available in the legend of Figure 1. Predicted amino-terminal signal peptide sequences were removed before alignment (names marked with asterisk). Pairs of tandem MMBLs present in a particular strain are marked with extensions (a) and (b).

## References

[b1] Allen HK, Trachsel J, Looft T, Casey TA (2014). Finding alternatives to antibiotics. Ann. N. Y. Acad. Sci.

[b2] Barreteau H, Bouhss A, Fourgeaud M, Mainardi JL, Touzé T, Gérard F (2009). Human- and plant-pathogenic *Pseudomonas* species produce bacteriocins exhibiting colicin M-like hydrolase activity towards peptidoglycan precursors. J. Bacteriol.

[b3] Church D, Elsayed S, Reid O, Winston B, Lindsay R (2006). Burn wound infections. Clin. Microbiol. Rev.

[b4] Ghequire MG, De Mot R (2014). Ribosomally-encoded antibacterial proteins and peptides from *Pseudomonas*. FEMS Microbiol. Rev.

[b5] Ghequire MG, Loris R, De Mot R (2012a). MMBL proteins: from lectin to bacteriocin. Biochem. Soc. Trans.

[b6] Ghequire MG, Li W, Proost P, Loris R, De Mot R (2012b). Plant lectin-like antibacterial proteins from phytopathogens *Pseudomonas syringae* and *Xanthomonas citri*. Environ. Microbiol. Rep.

[b7] Ghequire MG, Garcia-Pino A, Lebbe EK, Spaepen S, Loris R, De Mot R (2013a). Structural determinants for activity and specificity of the bacterial toxin LlpA. PLoS Pathog.

[b8] Ghequire MG, De Canck E, Wattiau P, Van Winge I, Loris R, Coenye T (2013b). Antibacterial activity of a lectin-like *Burkholderia cenocepacia* protein. Microbiologyopen.

[b9] Grinter R, Roszak AW, Cogdell RJ, Milner JJ, Walker D (2012). The crystal structure of the lipid II-degrading bacteriocin syringacin M suggests unexpected evolutionary relationships between colicin M-like bacteriocins. J. Biol. Chem.

[b10] Jakes KS (2014). Daring to be different: colicin N finds another way. Mol. Microbiol.

[b11] Jeganathan LP, Prakash L, Sivakumar N, Antony A, Alqarawi S, Prajna L (2014). Draft genome sequence of an invasive multidrug-resistant strain, *Pseudomonas aeruginosa* BK1, isolated from a keratitis patient. Genome Announc.

[b12] Johnson CL, Ridley H, Marchetti R, Silipo A, Griffin DC, Crawford L (2014). The antibacterial toxin colicin N binds to the inner core of lipopolysaccharide and close to its translocator protein. Mol. Microbiol.

[b13] Lam JS, Taylor VL, Islam ST, Hao Y, Kocincova D (2011). Genetic and functional diversity of *Pseudomonas aeruginosa* lipopolysaccharide. Front. Microbiol.

[b14] Ling H, Saeidi N, Rasouliha BH, Chang MW (2010). A predicted S-type pyocin shows a bactericidal activity against clinical *Pseudomonas aeruginosa* isolates through membrane damage. FEBS Lett.

[b15] Lipuma JJ (2010). The changing microbial epidemiology in cystic fibrosis. Clin. Microbiol. Rev.

[b16] McCaughey LC, Grinter R, Josts I, Roszak AW, Waløen KI, Cogdell RJ (2014). Lectin-like bacteriocins from *Pseudomonas* spp. utilise D-rhamnose containing lipopolysaccharide as a cellular receptor. PLoS Pathog.

[b17] Murphy K, Park AJ, Hao Y, Brewer D, Lam JS, Khursigara CM (2014). Influence of O polysaccharides on biofilm development and outer membrane vesicle biogenesis in *Pseudomonas aeruginosa *PAO1. J. Bacteriol.

[b18] Murugan N, Malathi J, Umashankar V, Madhavan HN (2014). Comparative genomic analysis of multidrug-resistant *Pseudomonas aeruginosa* clinical isolates VRFPA06 and VRFPA08 with VRFPA07. Genome Announc.

[b19] Parret AH, Schoofs G, Proost P, De Mot R (2003). Plant lectin-like bacteriocin from a rhizosphere-colonizing *Pseudomonas* isolate. J. Bacteriol.

[b20] Parret AH, Wyns L, De Mot R, Loris R (2004). Overexpression, purification and crystallization of bacteriocin LlpA from *Pseudomonas* sp. BW11M1. Acta Crystallogr. D Biol. Crystallogr.

[b21] Parret AH, Temmerman K, De Mot R (2005). Novel lectin-like bacteriocins of biocontrol strain *Pseudomonas fluorescens* Pf-5. Appl. Environ. Microbiol.

[b22] Pirnay JP, Bilocq F, Pot B, Cornelis P, Zizi M, Van Eldere J (2009). *Pseudomonas aeruginosa* population structure revisited. PLoS One.

[b23] Roy A, Kucukural A, Zhang Y (2010). I-TASSER: a unified platform for automated protein structure and function prediction. Nat. Protoc.

[b24] Ryder C, Byrd M, Wozniak DJ (2007). Role of polysaccharides in *Pseudomonas aeruginosa* biofilm development. Curr. Opin. Microbiol.

[b25] Stewart L, Ford A, Sangal V, Jeukens J, Boyle B, Kukavica-Ibrulj I (2014). Draft genomes of 12 host-adapted and environmental isolates of *Pseudomonas aeruginosa* and their positions in the core genome phylogeny. Pathog. Dis.

