# Supplementary figures and images for: O serotype-independent susceptibility of Pseudomonas aeruginosa to lectin-like pyocins
Source: Microbiologyopen. 2014 Sep 16;3(6):875–84. doi: 10.1002/mbo3.210 (PMC4263511; doi:10.1002/mbo3.210)

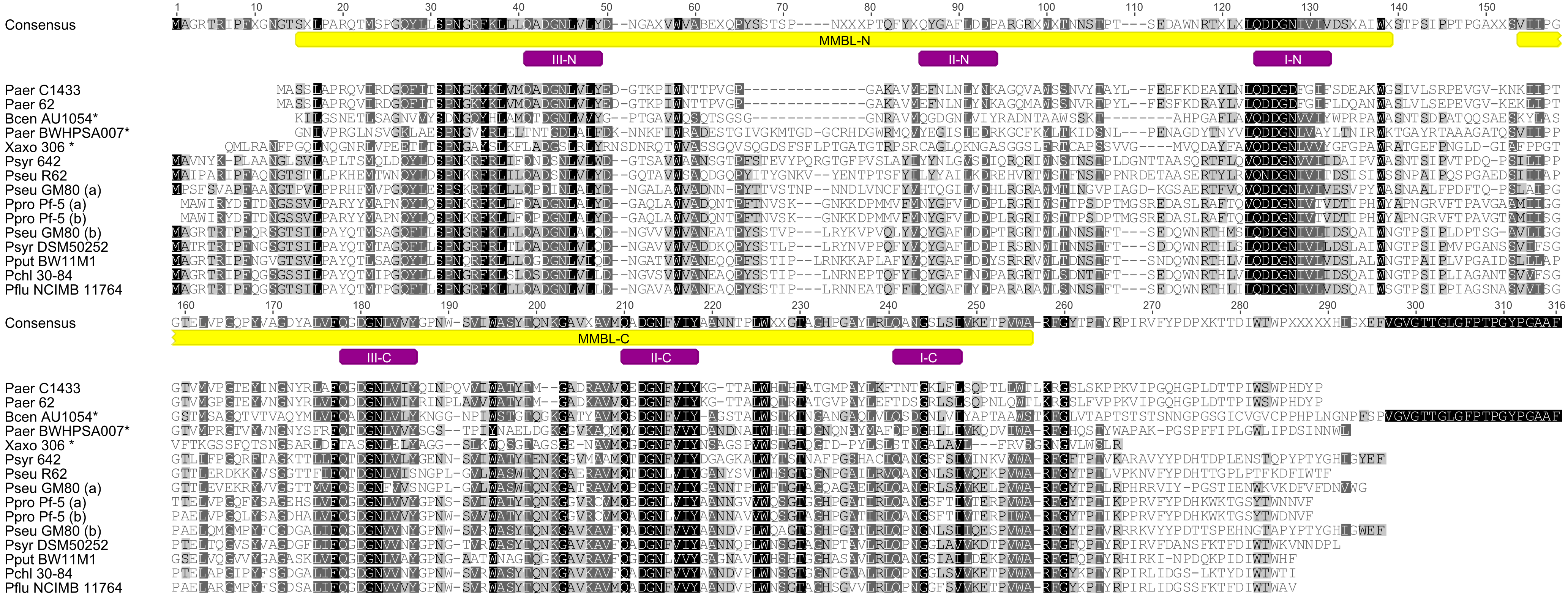

Supplement: Figure S1 — Multiple amino acid sequence alignment used to construct the phylogenetic tree of prokaryotic tandem-monocot mannose-binding lectin (MMBL) homologues (Fig. 1A). Sequence conservation is visualized by differential shading. The locations of MMBL modules in the amino-terminal domain (MMBL-N) and the carboxy-terminal domain (MMBL-C) are indicated by yellow boxes along the consensus sequence. The positions of the MMBL motifs constituting (putative) sugar-binding sites are marked by purple boxes. Aligned MMBL modules were used for the construction of the ML tree of Figure 1B (using the JTT matrix). Sequence designations are provided in the legend of Figure 1. [file mbo30003-0875-sd1.tif]
