# Supplementary material for: O serotype-independent susceptibility of Pseudomonas aeruginosa to lectin-like pyocins
Source: Microbiologyopen. 2014 Sep 16;3(6):875–84. doi: 10.1002/mbo3.210 (PMC4263511; doi:10.1002/mbo3.210)
Supplement: Table S2 — List of primers used in this study. [file mbo30003-0875-sd3.docx]

**Table S2.** List of primers used in this study.

| **Primer** | **Sequence^a^** | **Use** |
| --- | --- | --- |
| PGPRB-10029 | TGGCAGCAGCCAACTCAGCTT | Plasmid sequencing of pET28a constructs; forward primer |
| PGPRB-10030 | TATAGGCGCCAGCAACCGCA | Plasmid sequencing of pET28a constructs; reverse primer |
| PGPRB-10015 | TGGCTA**CATATG**GCTAGCTCTCTTGCTCCAC | Amplification of *pyoL2* (construction of pCMPG6210) |
| PGPRB-10016 | TGGCTA**CTCGAG**TTATGGATAGTCGTGGGGCCAAG | Amplification of *pyoL2* (construction of pCMPG6210) |
| PGPRB-10018 | TGGCTA**CATATG**GCAAGCTCTCTTGCTCCA | Amplification of *pyoL1* (construction of pCMPG6209) |
| PGPRB-10019 | TGGCTA**CTCGAG**TTATGGATAGTCGTGGGGCCAAG | Amplification of *pyoL1* (construction of pCMPG6209) |
| PGPRB-10054 | TGGCTA**CATATG**GGGAACATCGTACCACGTGGGTT | Amplification of *pyoL3* (construction of pCMPG6211) |
| PGPRB-10055 | TGGCTA**CTCGAG**TTACAGCCAGTTAGACACACTATCCGG | Amplification of *pyoL3* (construction of pCMPG6211) |

^a^ Restriction sites incorporated in primers are shown in bold: CATATG, NdeI; CTCGAG, XhoI.
